# Self-Assembling Short Peptide Carriers for Gene Delivery

**DOI:** 10.3390/ijms27083464

**Published:** 2026-04-12

**Authors:** Longyu An, Zhanyao Xu, Xiaoming Zhang

**Affiliations:** 1 School of Science, Minzu University of China, Beijing 100081, China; 25302682@muc.edu.cn (L.A.); xzhanyao@126.com (Z.X.); 2 Optoelectronics Research Centre, Minzu University of China, Beijing 100081, China

**Keywords:** short peptides, self-assembly, gene delivery, non-viral vectors, nanomedicine, supramolecular chemistry, biocompatibility

## Abstract

Gene therapy relies on safe and efficient delivery systems, yet traditional viral vectors and synthetic polymers often fail to meet these requirements due to immunogenicity and biocompatibility concerns. This review highlights self-assembling short peptides as a highly programmable and biocompatible non-viral platform uniquely positioned to overcome these translational bottlenecks. To provide a comprehensive overview of next-generation gene delivery, we systematically trace the trajectory from fundamental chemistry to clinical applications. First, we elucidate the supramolecular interactions and mechanisms driving peptide–nucleic acid co-assembly. Second, we outline concrete design strategies, detailing how sequence engineering and environmental responsiveness dictate the formation of optimized nanomorphologies. Third, we critically analyze how these nanocarriers navigate critical physiological and intracellular barriers, with a specific focus on cellular uptake, endosomal escape, and cargo release. Finally, we demonstrate the platform’s versatility in emerging frontiers, particularly mRNA vaccines and CRISPR/Cas9 gene editing. We conclude by identifying current obstacles to clinical translation and proposing future directions centered on multifunctional integration and stimuli-responsive design.

## 1. Introduction

### 1.1. The Rise in Gene Therapy and Delivery Challenges

For a long time, genetic and acquired diseases have posed significant challenges to human health. In the field of oncology, traditional therapies are limited by severe side effects, driving the need for targeted drug delivery systems with high specificity and low toxicity [1]. In metabolic diseases, given the massive patient population and poor compliance with conventional injections, there is an urgent demand for long-acting and orally administered therapies. Furthermore, in cardiovascular and neurodegenerative diseases, existing medications often carry substantial side effects or merely provide symptomatic relief, underscoring the critical need for curative, targeted therapies capable of crossing physiological barriers [2]. Ultimately, traditional therapies relying on small-molecule or protein-based drugs typically only manage or alleviate disease symptoms, failing to provide durable solutions or definitive cures [3].

**Figure 1:**
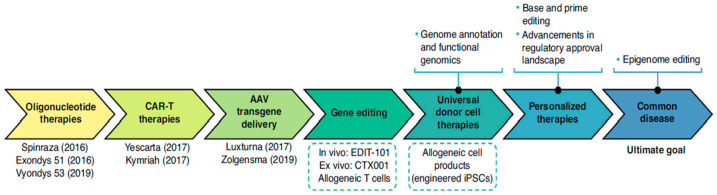
In the past, CAR-T and AAV delivery therapies made great strides in treating uncommon disorders and certain types of cancer. At the moment, the industry is using gene editing and universal donor cell technologies to fix technical problems and make things easier to get to. In the future, improved epigenome editing will be used to make medicine more individualized, with the ultimate goal of beating common diseases. Adapted from Ref. [9].

Heralded as the third medical revolution following small-molecule therapeutics and antibody-based interventions, gene therapy aims to rectify genetic defects at their fundamental origin via the introduction, editing, or silencing of specific nucleotide sequences [4,5,6]. Since the conceptual inception of gene therapy in the 1970s [7,8], the maturation of technologies such as CRISPR/Cas9, siRNA, and mRNA has precipitated a paradigm shift. The field has evolved from rudimentary gene fragment replacement into a sophisticated nucleic acid therapeutic system capable of precise genomic modification and post-transcriptional regulation [9]. Consequently, the therapeutic scope has rapidly expanded beyond initial applications in monogenic disorders, such as spinal muscular atrophy and retinopathy [10], to encompass highly complex pathologies, including malignancies, neurodegenerative disorders, and metabolic syndromes [11,12,13,14,15,16,17] (Figure 1). Such therapies hold the promise of delivering durable therapeutic effects and, in certain cases, even achieving complete cures. For patients and their families facing severe, life-threatening diseases that currently lack effective treatment options, these therapies offer the hope of a new lease on life.

Beyond its applications in human healthcare, gene therapy has expanded into fields such as agriculture, disease control, and environmental management. In agriculture, technologies like RNA interference are increasingly being used to silence specific endogenous or viral genes. This approach bypasses the slow process of traditional breeding, rapidly enhancing yields and animal disease resistance [18]. In terms of disease control, researchers are developing self-disseminating vaccines within wildlife populations to prevent outbreaks of viruses such as SARS-CoV-2, Ebola, and Nipah at their source [19]. Meanwhile, in environmental management, researchers are using gene therapy to help native soil bacteria acquire new genes, thereby accelerating the degradation of pollutants [20]. In summary, these cross-disciplinary applications fully demonstrate the immense potential of gene therapy.

**Figure 2:**
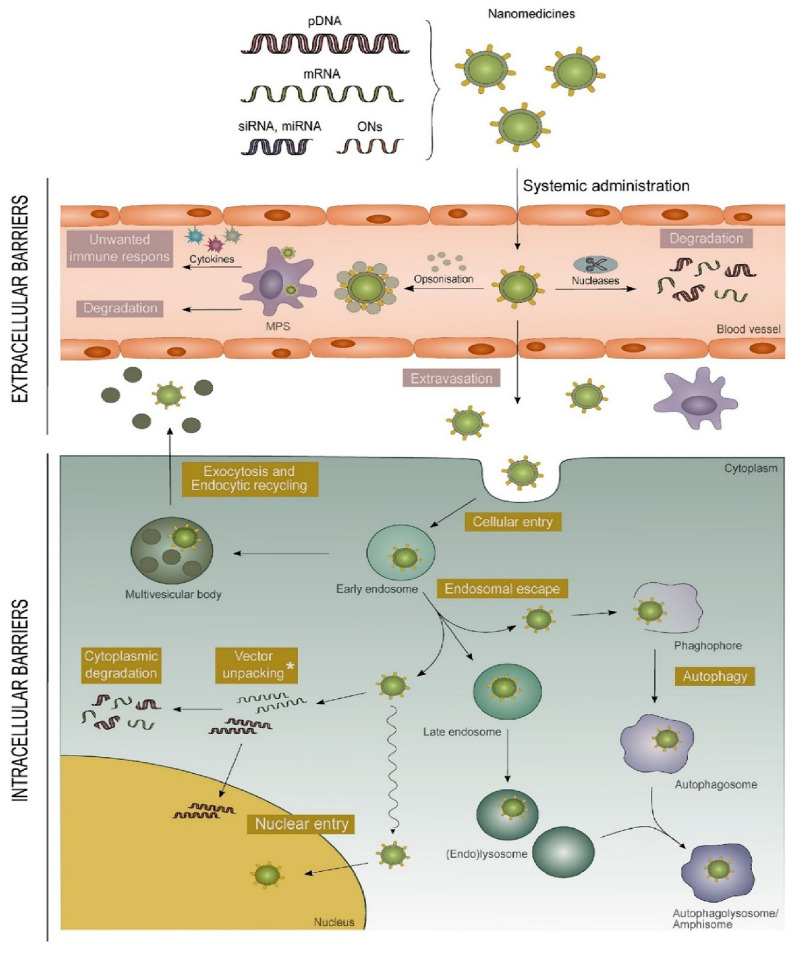
Schematic illustration of the extracellular and intracellular barriers hindering systemic gene delivery. Adapted from Ref. [1]. * Vector unpacking could also occur inside nucleus or endosomes.

Even if nucleic acid molecular design is getting more advanced, the effectiveness of a treatment still depends on the structural integrity and delivery of the genetic payload to the target site. So, getting accurate, high-efficiency targeted delivery is still the fundamental problem that is slowing down clinical translation. A perfect gene delivery vector needs to be carefully thought out so that it can get past a number of tough physiological barriers [21]. Initially, the vector must evade rapid clearance by the immune system [22]. Upon reaching the target tissue, it encounters interfacial barriers presented by the plasma membrane and the dense glycoprotein network, necessitating cellular entry via endocytosis or direct membrane fusion [23]. Subsequently, the vector must execute endosomal escape prior to acidification and lysosomal enzymatic degradation to reach the cytoplasm [24]. Finally, the vector is required to undergo disassembly at the target site to liberate the therapeutic cargo. In the specific case of DNA-based therapeutics, the cargo must further traverse the nuclear pore complex to enter the nucleus [25] (Figure 2).

Aside from physiological barriers, production economics constitute a significant factor influencing the broad availability of gene treatments. Many gene therapies for uncommon diseases cost millions of dollars, which is mostly because of the high costs of making vectors and managing a complicated supply chain. Consequently, the advancement of delivery vectors that synergistically combine high-efficiency barrier penetration with cost-effective, scalable manufacturing presents considerable promise and has attracted major focus as a primary goal in global biomedical research [26].

### 1.2. Limitations of Existing Gene Delivery Vectors

Through eons of evolutionary pressure, viruses have engineered sophisticated mechanisms for efficient host cell infiltration. Consequently, viral vectors have historically constituted the cornerstone of clinical gene therapy [27,28]. However, their intrinsic nature as exogenous pathogens introduces severe biosafety concerns [29]. The inherent immunogenicity of viral capsids not only precludes repeated administration but also poses a risk of inducing fulminant inflammatory responses [28,30]. Furthermore, integrating viral vectors carries the latent peril of insertional mutagenesis and oncogenesis [27].

To avoid the safety risks that come with viral methods, scientists have switched to synthetic and inorganic non-viral vectors, which are mostly made up of cationic liposomes, polymeric systems and inorganic nanomaterials such as nanoclays.

The clinical success of Lipid Nanoparticles (LNPs) in COVID-19 mRNA immunization highlights their promise as nucleic acid delivery systems; however, their use in systemic gene therapy is still problematic. Primarily, cationic lipids promote cellular internalization through electrostatic interactions with anionic cell membranes; nevertheless, this strong attraction frequently undermines membrane integrity [31]. Secondarily, while surface modification with polyethylene glycol (PEG) extends circulatory half-life, it paradoxically creates a steric barrier that inhibits cellular uptake and endosomal escape [32]. Additionally, frequent administration of PEGylated lipids might induce the formation of anti-PEG antibodies, leading to the “accelerated blood clearance” (ABC) phenomenon [33,34]. Finally, during systemic administration, the absence of specific targeting ligands renders LNPs susceptible to rapid sequestration by the reticuloendothelial system and passive accumulation in the liver and spleen. This results in suboptimal transduction efficacy in non-hepatic tissues and severe off-target toxicity [21,35].

Similarly, cationic polymers, epitomized by polyethyleneimine (PEI), were initially heralded for their exceptional endosomal escape capability, attributed to the “Proton Sponge Effect.” However, this mechanism relies on high cationic charge density, which inevitably induces severe membrane disruption [36,37]. A critical dichotomy exists in PEI formulations: high-molecular-weight PEI offers superior transfection efficiency but exhibits significant cytotoxicity and poor biodegradability, whereas low-molecular-weight PEI possesses a favorable safety profile but fails to effectively condense DNA [31,36]. Compounded by a lack of intrinsic targeting specificity and biological degradability, the long-term safety profile of PEI-based systems remains precarious.

While the early success of nanoclays in drug delivery and gene therapy underscores their tremendous potential as non-viral nucleic acid vectors, their application in systemic gene therapy remains hindered by several key challenges [38,39,40]. Although surface charges and interlayer cation bridging enable nanoclays to effectively bind and internalize nucleic acids, this intense electrostatic attraction—coupled with unmodified active sites and sharp physical edges—often compromises cell membrane integrity, causing direct membrane damage and cytotoxicity [39,40]. Additionally, because certain nanoclays act as persistent nanomaterials, repeated administration may pose a risk of long-term in vivo bioaccumulation. Furthermore, nanoclay-nucleic acid complexes often exhibit instability within complex physiological charge environments. Lastly, due to their high aspect ratio, nanoclays may provoke non-specific cellular over-uptake and potential off-target toxicity during administration [40].

In summary, current vectors face inherent trade-offs: viral systems provide high transduction efficiency but carry safety risks, while synthetic alternatives are safer but limited by cytotoxicity and poor intracellular trafficking. Likewise, inorganic vectors like nanoclays, despite their potential, remain challenged by physiological instability and long-term bioaccumulation. (Table 1).

### 1.3. Carrier Design Principles

First, the carrier must effectively condense and encapsulate genetic material via non-covalent interactions to protect it from rapid nuclease degradation [8,21]. This creates a binding paradox: the carrier must tightly complex with the gene to survive the bloodstream, yet loosely enough to unpack and release its cargo inside target subcellular compartments. Once in the systemic circulation, the nanoparticles must rely on surface modifications, such as PEGylation, to evade clearance by the immune and mononuclear phagocyte systems [1,8,21]. Subsequently, they utilize surface-conjugated targeting ligands to achieve specific endocytosis into the target tissues [22]. Upon entering the target cell, the carrier must be capable of disrupting the endosomal membrane, enabling the genetic material to successfully escape into the cytoplasm to ultimately complete the highly demanding process of subcellular localization [1,21]. Finally, the carrier must ensure exceptional biocompatibility and low cytotoxicity, offer precise modulation of immunogenicity tailored to specific application scenarios, and possess the capacity for standardized, large-scale manufacturing [22] (Table 2).

All successfully engineered self-assembling nanosystems exhibit highly consistent commonalities in their underlying physicochemical mechanisms. They are fundamentally built upon a precise dynamic balance of various weak, non-covalent forces, including hydrogen bonding, hydrophobic interactions, electrostatic interactions, and π-π aromatic stacking [1,42]. At the same time, modern delivery vehicles are generally designed with “smart” environmental stimuli-responsiveness, capable of sensitively detecting minute changes in the target microenvironment to trigger structural transformations and achieve on-demand release [23,42]. Beyond biophysical and cellular-level constraints, all candidate gene delivery systems must ultimately meet the fundamental requirements for clinical translation. This means that from the very beginning of the design process, new systems must satisfy several universal characteristics: the materials must possess excellent biodegradability, and their chemical synthesis processes must be amenable to standardized, large-scale production suitable for human use [23]. Concurrently, the carrier system must demonstrate a sufficiently broad therapeutic window, maintain strict batch-to-batch consistency, and ensure that throughout the repeated dosing cycles required in clinical settings, it does not trigger strong host immune responses that could lead to diminished efficacy or serious adverse events [7,22] (Table 3).

### 1.4. The Emergence of Short Peptides as Gene Delivery Vectors

Peptide-based self-assemblies are becoming the most important area of gene delivery research because viral vectors are dangerous, and synthetic ones are toxic. Peptides are usually defined as oligomeric sequences made up of 5 to 30 amino acids. They fill an important gap by combining the biological functions of proteins with the chemical control of small molecules [2,3,44]. Peptide self-assemblies strike an optimal balance between functionality and safety at the molecular level, thereby effectively resolving the recalcitrant limitations of preceding vector generations.

First and foremost, peptide vectors exhibit inherently superior biocompatibility and safety profiles [45]. Peptides are made up of amino acids that are found in nature, which is very different from synthetic polymers. This makes sure that the waste products from their metabolism are not harmful, which gets rid of the risk of long-term cumulative toxicity. Additionally, their immunogenicity is considerably reduced in comparison to viral vectors, thereby creating a solid safety framework for clinical gene therapy [25,46,47].

Secondly, peptides demonstrate unparalleled sequence programmability and synthetic accessibility. Researchers can orchestrate vector function with high precision at the molecular scale [2,3]. Regarding fabrication, Solid-Phase Peptide Synthesis (SPPS) has obviated the need for cumbersome biological culture systems. This technology facilitates highly automated, scalable production while permitting the incorporation of non-natural amino acids and site-specific modifications, thereby substantially expanding the repertoire of functional diversity.

**Figure 3:**
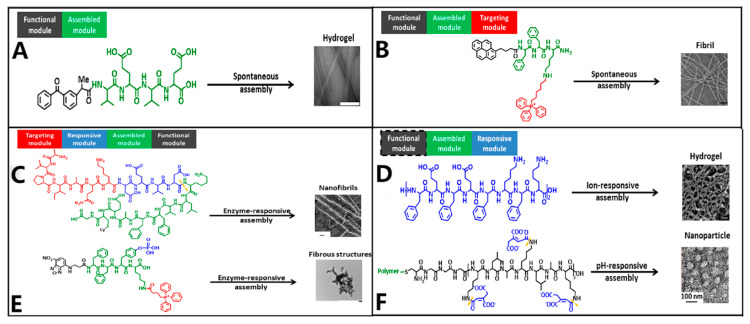
Modular design and self-assembly strategies of peptide-based nanomaterials. The schematic illustrates the formation of hydrogels (**A**,**D**), fibrils (**B**,**C**,**E**), and nanoparticles (**F**) through spontaneous or stimuli-responsive pathways using functionalized peptide blocks. Adapted from Ref. [3].

Lastly, peptides have their own special properties that make them able to self-assemble. When certain peptide sequences are exposed to certain environmental stimuli, they spontaneously form hierarchical structures that can take the form of nanofibers, vesicles, hydrogels, or micelles. This method not only protects nucleic acid payloads by tightly wrapping them up and using enzymes, but it also lets us mimic the way extracellular matrix (ECM) nanostructures work. These features help cells stick together and keep releasing drugs, which makes these systems especially useful for localized delivery and gene therapy combined with tissue engineering [46,48,49,50] (Figure 3).

## 2. Interactions Between Short Peptides and Genetic Material and Self-Assembly Mechanisms

### 2.1. Intermolecular Forces Driving Self-Assembly

The co-assembly of short peptides with nucleic acids surpasses mere physical mixture, embodying a complex phenomenon regulated by both thermodynamic and kinetic factors. A combination of non-covalent interactions, such as electrostatic attraction, hydrogen bonding networks, hydrophobic effects, and π-π stacking, controls this process. To make efficient delivery vectors, we need to know the physicochemical nature of these forces and how they relate to each other in the assembly [51,52,53] (Table 4).

#### 2.1.1. Electrostatic Interactions

Electrostatic interaction is the main force that holds cationic peptides to the anionic nucleic acid scaffold. At physiological pH, the phosphate backbone of nucleic acids has a high negative charge density. On the other hand, rationally designed peptides usually have more basic amino acid residues that can be protonated [56,59,60]. This association precipitates a pronounced charge neutralization effect; upon neutralizing approximately 90% of the backbone charge, intramolecular repulsion diminishes significantly, triggering the rapid collapse of the payload into compact nanostructures [55]. Thermodynamically, this condensation is an entropy-driven process, mediated by the displacement of counterions originally bound to the DNA surface [57]. While incomplete neutralization yields structurally loose complexes, effective condensation generates uniform nanostructures characterized by surface charge reversal [61]. This resultant cationic surface effectively mitigates inter-particle aggregation, thereby preserving colloidal stability within biological environments [62]. However, excessive electrostatic affinity may impede cargo release intracellularly, necessitating a precise trade-off between condensation capacity and release efficiency in sequence design [63].

#### 2.1.2. Hydrogen Bonding

If electrostatics are the “glue,” hydrogen bonding is the “scaffold” that holds the assembly together and controls its internal shape and secondary structure. The strong directionality of hydrogen bonds creates spatial limits that force peptide chains to change from thermodynamically unstable random coils to ordered secondary motifs, like β-sheets, by changing the angles of the backbone torsion. In these structures, a network of hydrogen bonds forms between the backbone amides of adjacent chains that are perpendicular to the fiber axis. This makes it easier to stack the chains into high-aspect-ratio nanofibers or nanotapes [57,62,64].

**Figure 4:**
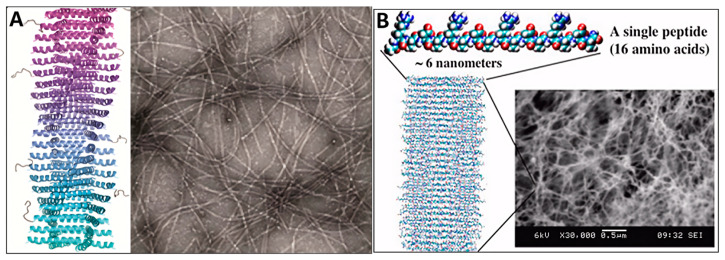
(**A**) Intramolecular hydrogen bond-driven helical assembly. The figure displays the process where α-helix pairs assemble into right-handed superhelical nanofibers under the guidance of hydrogen bonds, visually illustrating the decisive role of hydrogen bonds as the “skeleton” of the internal geometric configuration. (**B**) Construction of supramolecular skeletons via intermolecular hydrogen bonds. This figure illustrates how RADA16 peptides assemble from the single-molecule level into a thermodynamically stable supramolecular hydrogel scaffold through the synergy of hydrogen bond networks and hydrophobic effects. Adapted from Ref. [65].

The kinetics of self-assembly rely essentially on the competition between “peptide-peptide” and “peptide-solvent” hydrogen bonding [58]. Constructing a thermodynamically stable supramolecular framework requires the synergistic exclusion of water molecules from the core via hydrophobic effects, thereby eliminating competitive disruption by the solvent [57]. And enhancing core hydrophobicity significantly accelerates this desolvation process [58]. Consequently, intermolecular hydrogen bonding predominantly yields nanofibers or nanoribbons, whereas intramolecular bonding favors the initial formation of helical bundles, which subsequently assemble into micelles, vesicles, or superhelical fibers [56,65] (Figure 4).

#### 2.1.3. Hydrophobic Interactions

**Figure 5:**
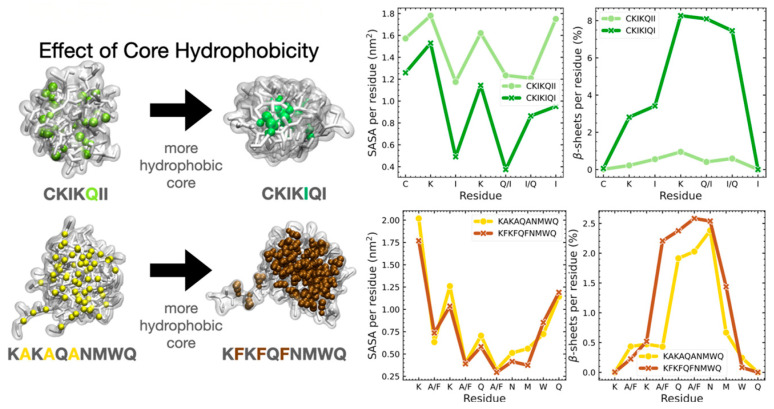
Simulation results show that enhancing core hydrophobicity by introducing strongly hydrophobic residues significantly reduces the solvent accessible surface area and increases β-sheet content. The spheres illustrate the compact packing of hydrophobic residues within the fibril cross-sectional core, corroborating the dominant role of the hydrophobic core in driving assembly and maintaining structural rigidity. Adapted from Ref. [58].

Hydrophobic interaction functions as the principal driver for the self-assembly of amphiphilic peptides into core–shell architectures, such as micelles and vesicles. Governed by the hydrophobic effect, non-polar amino acid residues aggregate in aqueous media to minimize interfacial exposure, thereby reducing the energetic penalty associated with disrupting the solvent’s hydrogen bond network [66,67]. The strength of this force directly affects the critical micelle concentration (CMC) and the kinetic stability of the assembly. Peptides with moieties that are more hydrophobic usually have lower CMCs. The creation of a hydrophobic core not only helps with assembly, but it also creates a separate space for the co-encapsulation of hydrophobic therapeutics or adjuvants [58,68,69]. Furthermore, the specific spatial patterning of hydrophobic residues modulates the resultant morphological topology [54,70] (Figure 5).

#### 2.1.4. π-π Stacking Interactions

**Figure 6:**
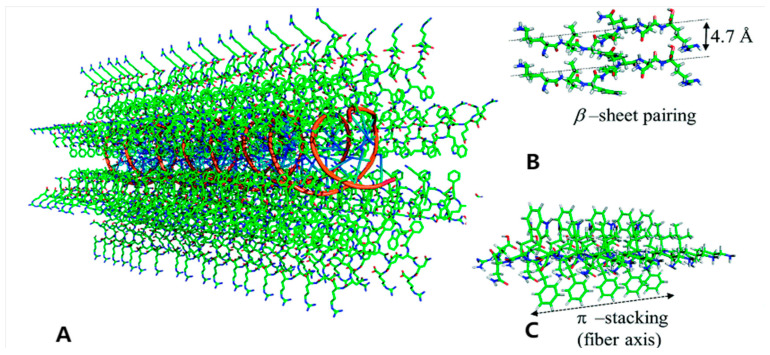
Hierarchical assembly and key interaction mechanisms of aromatic peptide fibrils: (**A**) Schematic of the rigid fibrillary scaffold formed by peptide self-assembly, exhibiting high structural stiffness. (**B**) Intermolecular backbone interactions showing β-sheet pairing stabilized by hydrogen bond networks. (**C**) Detailed view of π-π stacking interactions along the fiber axis. Aromatic residues form dense, ordered arrays that act as “steric zippers.” This interlocking mechanism excludes water molecules from the core and locks the inter-sheet distance, providing the structural basis for the assembly’s enhanced mechanical rigidity and resistance to environmental erosion. Adapted from Ref. [56].

Aromatic residues play a multifaceted role in peptide self-assembly, functioning not merely as hydrophobic fillers but as critical stabilizers via specific stacking modes. The unique electronic quadrupole moments of their side-chain aromatic rings dictate anisotropic binding interactions [71,72]. π-π stacking confers stability superior to that of simple hydrogen bond networks by constructing dense aromatic clusters. Simulation studies elucidate that this dense packing forms an interlocking mechanism analogous to a “steric zipper,” which significantly elevates β-sheet content and locks interlayer spacing. This interlocking effect endows nanotubes with exceptional mechanical rigidity and thermal stability. Moreover, the resulting dense network restricts protease access and compensates for electrostatic screening in high-ionic-strength environments; under conditions where electrostatic forces are attenuated, aromatic stacking—often in concert with cation-π interaction—emerges as the dominant force maintaining structural integrity [73] (Figure 6).

### 2.2. Typical Self-Assembled Nanostructures

**Figure 7:**
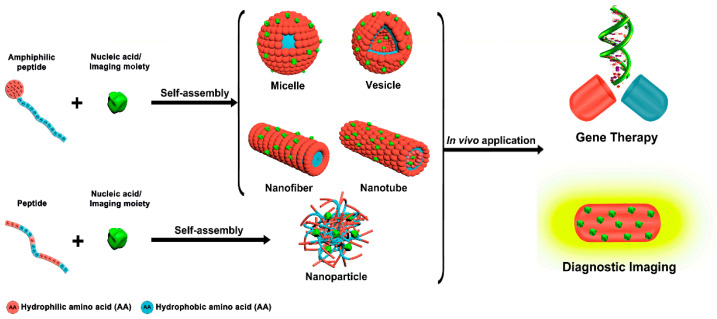
Schematic of amphiphilic peptide-nucleic acid co-assembly. Guided by peptide packing parameters and physicochemical properties, the process yields 0D or 1D architectures. These nanostructures facilitate efficient nucleic acid encapsulation for applications in gene therapy and diagnostic imaging. Furthermore, the physical entanglement of these one-dimensional nanofibers can further evolve into three-dimensional hydrogel networks. Adapted from Ref. [56].

The ultimate morphology of peptide self-assemblies is governed by the intricate interplay of molecular amphiphilicity, charge distribution, and packing parameters. Within the realm of gene delivery, three distinct architectural classes have garnered substantial attention: one-dimensional nanotubes/nanofibers, zero-dimensional nanovesicles/micelles, and three-dimensional hydrogel networks. Each topological configuration exhibits unique advantages and limitations regarding size effects, cargo preservation capabilities, and intracellular transport mechanisms (Figure 7).

#### 2.2.1. Nanotubes/Nanofibers

One-dimensional nanostructures, distinguished by elevated aspect ratios, arise from the anisotropic propagation of peptide monomers. The FF dipeptide forms hollow nanotubes by stacking aromatic and amphiphilic molecules on top of each other. The walls of the tubes are made up of peptide multilayers that surround a hydrated lumen. On the other hand, surfactant-like peptides like A_6_K tend to form solid nanofibers, with hydrophobic tails hidden in the core and hydrophilic headgroups on the outside [66,68,74].

Given that pristine hollow nanotubes like FF often lack intrinsic affinity for genetic cargo, a prevalent engineering strategy involves the incorporation of cationic residues to facilitate electrostatic nucleic acid loading via high-density positive charges [75]. This topological configuration not only accommodates an exceptionally high gene payload but also mimics the fibrillar nature of the extracellular matrix, thereby enhancing fluid dynamics and cellular uptake [76,77].

Crucially, while excessive aspect ratios in traditional inorganic materials frequently precipitate “frustrated endocytosis”, inducing lysosomal membrane permeabilization and cytotoxicity [78], certain self-assembled peptide nanofibers exhibit a distinct “dimensional advantage” absent of such virulence [77,79]. To reconcile the trade-off between surface area for gene loading and specific endocytic efficiency, fiber lengths are often modulated to 100–200 nm via kinetic control or sonication-induced fragmentation, thereby achieving an optimal balance between delivery potency and biosafety [80].

#### 2.2.2. Nanovesicles/Micelles

Spherical assemblies represent the most established paradigm in drug delivery, offering an isotropic geometry that inherently aligns with hydrodynamic behavior in biological fluids.

Zero-dimensional architectures primarily encompass micelles and nanovesicles. Micelles typically adopt a solid “core–shell” configuration, where the hydrophobic interior serves to solubilize non-polar therapeutics. In contrast, vesicles comprise a hollow bilayer membrane formed by the “tail-to-tail” alignment of monomers; their hydrophilic interior provides an ideal compartment for encapsulating macromolecular payloads such as nucleic acids or proteins. Unlike high-aspect-ratio nanotubes that may linger at the cell surface, the structural transition to vesicular morphology significantly accelerates cellular internalization [81,82]. However, given the thermodynamic propensity of short peptides to form long-range ordered nanofibers [83,84], engineering discrete vesicular morphologies often necessitates physical intervention—such as ultrasonication—to energetically manipulate the assembly pathway [70,82,85,86,87,88,89].

#### 2.2.3. Hydrogels

A three-dimensional hydrogel network, exhibiting water content exceeding 99%, is established when peptide nanofibers achieve a critical concentration threshold, leading to physical entanglement or crosslinking.

These supramolecular hydrogels rely on hierarchical assembly driven by non-covalent interactions to construct a micro-topological platform optimized for gene entrapment. The process initiates with the hydrophobic collapse of amphiphilic monomers into a thermodynamically stable β-sheet [90], which subsequently stacks into nanofibers or nanoribbons [84,91]. Upon exposure to physiological ionic strength, the screening of surface electrostatic repulsion triggers a sol–gel phase transition, instantaneously stabilizing the crosslinked network [84,90,92]. The cardinal advantages of these hydrogels lie in their shear-thinning behavior and thixotropic self-healing capabilities [93,94]. Furthermore, the dense fibrous matrix creates a steric barrier against nuclease infiltration, significantly prolonging the biological half-life of the cargo. Release kinetics are governed by a synergistic mechanism of Fickian diffusion and scaffold erosion, facilitating sustained delivery [95,96] (Table 5).

### 2.3. Strategies for Regulating the Assembly Process

Engineering high-performance delivery vectors predicates on the precise regulation of the self-assembly trajectory. This modulation is orchestrated via a bipartite strategy: intrinsic molecular sequence design and extrinsic environmental intervention.

#### 2.3.1. Peptide Sequence Design

**Figure 8:**
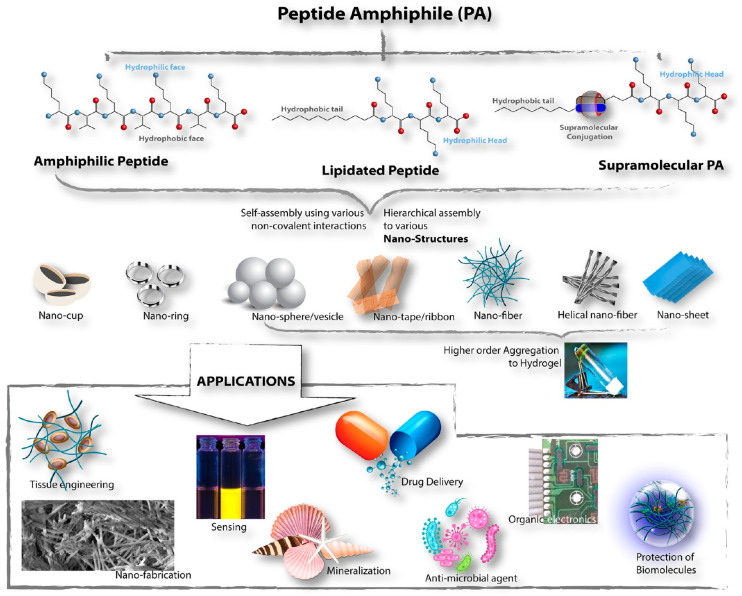
Sequence design and hierarchical assembly of Peptide Amphiphiles (PAs). Rational design of amino acid sequences yields diverse nanostructures (micelles, fibers, and vesicles) for various biomedical applications. Adapted from Ref. [99].

The amino acid sequence dictates the self-assembly behavior of short peptides. Rational design allows for the pre-programming of morphological topology, charge density, and functional characteristics [42,76,93,97]. For surfactant-like peptides, the resultant morphology is predictable via the critical packing parameter *P*. Theoretically, a value of *P* < 1/3 favors the formation of spherical micelles; 1/3 < *P* < 1/2 yields cylindrical micelles or nanofibers; and 1/2 < *P* < 1 predisposes the system toward bilayer vesicles [70]. However, the strong directionality of the peptide backbone hydrogen bonding often complicates this geometric prediction, yielding architectures more intricate than simple surfactant models suggest. Beyond geometry, the specific constitution of amino acids modulates the physicochemical properties of the assembly [98,99,100], whereas the specific sequential arrangement dictates the structural topology [76] (Figure 8).

#### 2.3.2. Environmental Factors

Post-synthesis, manipulating the solvent microenvironment serves as a potent trigger to induce assembly or actuate structural transitions.

Solution pH directly remodels the intermolecular electrostatic equilibrium by modulating the protonation state of amino acid side chains [100]. This mechanism hinges on ionizable residues [101,102]. When the environmental pH crosses the residue’s pKa threshold, the surface charge distribution inverts, shifting the balance of non-covalent forces and precipitating structural reconfiguration [100,103].

**Figure 9:**
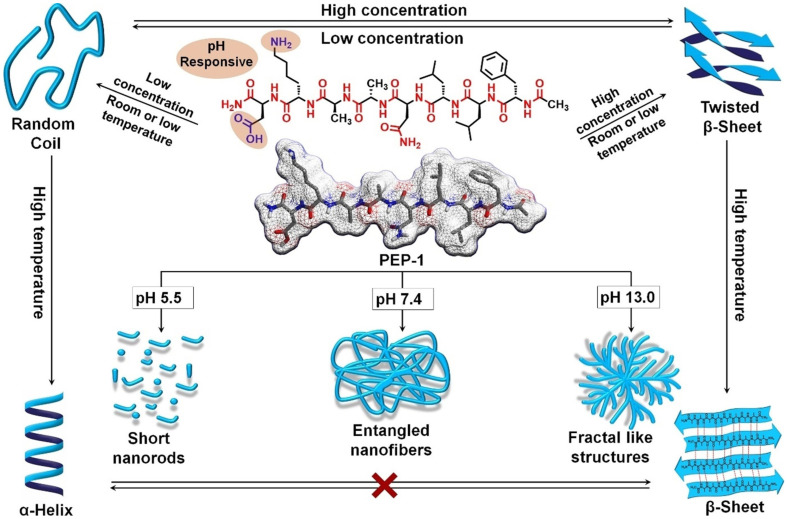
Regulation mechanisms of environmental factors on peptide self-assembly. The diagram illustrates how variations in pH, temperature, and concentration drive conformational transitions, resulting in distinct morphologies such as nanorods, fibers, or fractal structures. Adapted from Ref. [103].

Temperature modulates the equilibrium between solvation effects and weak intermolecular interactions by altering the system’s thermal energy. While elevated temperatures generally disrupt directional hydrogen bonds, they can—counterintuitively—potentiate hydrophobic interactions within a specific range due to the entropic gain derived from water release [93]. Thermal annealing is pivotal for manipulating kinetic versus thermodynamic states. Rapid cooling often kinetically traps peptide chains in a “frozen” disordered state, whereas controlled annealing permits molecules to surmount energy barriers and rearrange into the thermodynamic minimum [103]. Furthermore, tuning the sol–gel transition temperature to align with physiological conditions is critical for biomedical applicability [97] (Figure 9).

Ionic strength governs assembly kinetics by tuning the range of electrostatic interactions. In charged peptide amphiphiles, the introduction of salts compresses the electrical double layer, inducing Debye screening. This effect lowers the electrostatic repulsion barrier, allowing hydrophobic effects and hydrogen bonding to dominate, thus triggering fibrillation or gelation. However, excessive charge screening at physiological ionic strengths can compromise colloidal stability, leading to uncontrolled aggregation [76].

Ultrasonication facilitates the reorganization of disordered aggregates into thermodynamically stable ordered structures via acoustic cavitation—generating transient local hotspots of high temperature/pressure—and intense shear forces [93]. Furthermore, physical shearing fragments entangled fiber networks into short nanorods; this “fragmentation-reassembly” cycle accelerates the evolution toward the thermodynamic steady state, eliminating macroscopic aggregates and significantly reducing the polydispersity index. High-intensity ultrasound ensures size homogeneity, a parameter instrumental for consistent biological performance.

**Figure 10:**
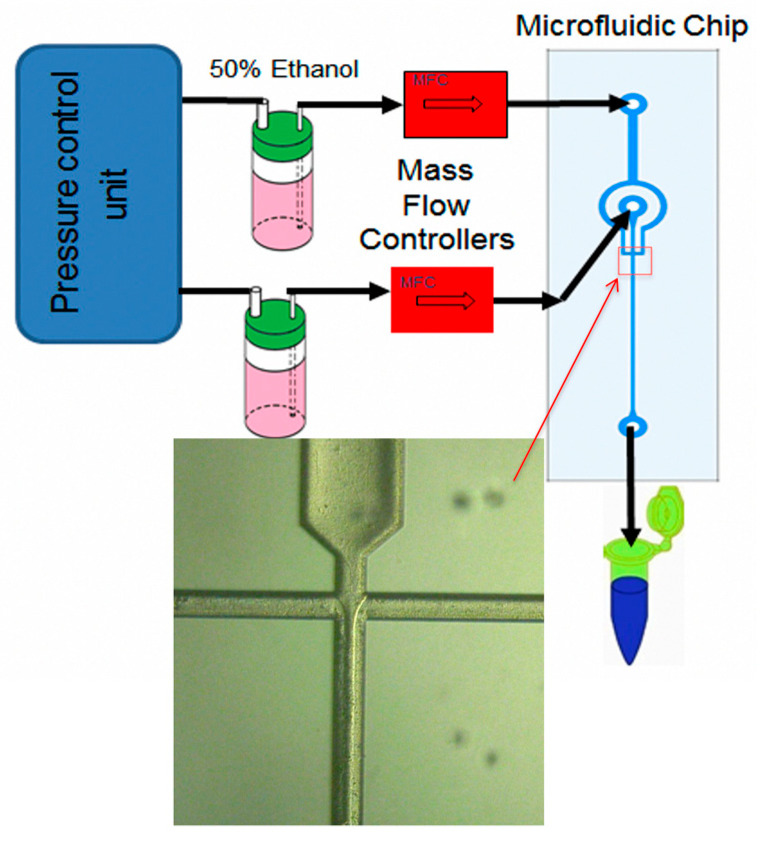
Schematic of microfluidic-assisted solvent exchange. The process utilizes hydrodynamic focusing to precisely control mixing, enabling the controllable preparation of peptide nanoparticles. Adapted from Ref. [104].

The solvent switch method precisely regulates nucleation via abrupt microenvironmental shifts. The protocol involves dissolving peptides in a “good” solvent followed by the introduction of a “poor” solvent. The resultant polarity shift precipitates a drastic reduction in solubility, triggering hydrophobic collapse, which subsequently extends into supramolecular structures via hydrogen bonding and π-π stacking. The solvent volumetric ratio dictates the final topology [99]. Crucially, the rate of exchange determines the assembly pathway; microfluidic-assisted rapid solvent exchange is particularly advantageous for engineering uniform spherical nanoparticles [104] (Figure 10).

## 3. Intracellular Delivery of Short Peptide Vectors

Distinct from liposomal or polymeric counterparts, peptide-based vectors represent the vanguard of non-viral gene delivery, distinguished by the rational design of amino acid sequences that permit precise modulation of interfacial interactions with plasma, endosomal, and nuclear membranes. The intracellular trajectory of these vectors comprises three critical phases: Cellular Uptake, Endosomal Escape, and Intracellular Release/Nuclear Import. This section provides a comprehensive mechanistic overview of this cascade.

### 3.1. Cellular Uptake

#### 3.1.1. Cellular Uptake Pathways

CME imposes strict size constraints on cargo [105]. Upon specific binding of the vector to surface receptors, the intracellular domain of the receptor recruits the adaptor protein complex 2, which orchestrates the polymerization of clathrin on the cytosolic leaflet, driving membrane invagination into clathrin-coated pits. Subsequently, dynamin assembles at the pit neck, utilizing energy derived from GTP hydrolysis to constrict and execute membrane scission, releasing the clathrin-coated vesicle [106]. Following uncoating, the vesicle fuses with early endosomes, where the luminal pH progressively drops [107]. This acidic milieu, coupled with high hydrolase activity in lysosomes, necessitates that the vector system possesses a robust “endosomal escape” capability to prevent payload degradation [108].

CvME is contingent upon lipid rafts—membrane microdomains enriched in cholesterol and sphingolipids that confer rigidity and serve as anchoring sites for the driver protein, Caveolin. Caveolin induces curvature by inserting into the inner membrane leaflet, functioning synergistically with the cytosolic coat protein Cavin to stabilize characteristic flask-shaped invaginations [105]. While CvME exhibits inherent spatial constraints that limit the uptake of large polymer aggregates, the kinetic process is triggered by vector-induced receptor clustering in lipid rafts. This precipitates caveolin oligomerization and Src kinase-mediated tyrosine phosphorylation. Subsequently, the GTPase dynamin assembles at the neck to execute dynamin-dependent scission [79]. Although distinct from CME, caveolar vesicles may also fuse with early endosomes, potentially routing cargo through late endosomes to lysosomes [32,109].

**Figure 11:**
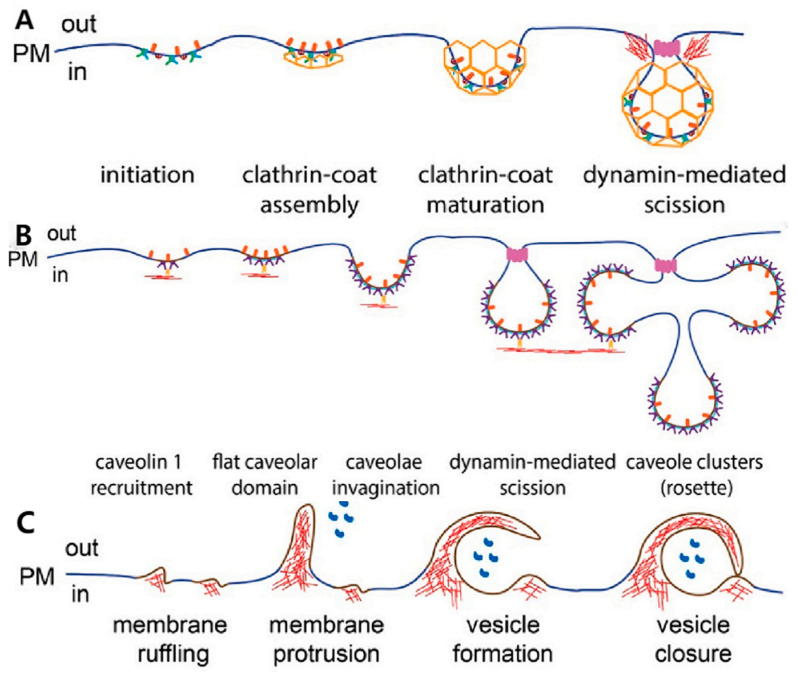
Schematic representation of major endocytic pathways: (**A**) Clathrin-mediated endocytosis. (**B**) Caveolae-mediated endocytosis. (**C**) Macropinocytosis. Adapted from Ref. [1].

In contrast to the high-fidelity receptor-ligand recognition of CME, macropinocytosis is an actin-driven, high-capacity, non-specific mechanism facilitating the “bulk uptake” of extracellular fluids and solutes [79,105]. Mechanistically, specific stimuli activate Ras, Src kinases, and Rho GTPases, alongside the modulation of sub-membranous pH by Na^+^/H^+^ exchangers and phosphoinositide 3-kinase activity [79,110]. These signaling cascades induce vigorous polymerization and depolymerization cycles of actin filaments, forcing the plasma membrane to protrude and form irregular membrane ruffles. As these ruffles collapse or fuse, significant volumes of extracellular fluid are physically entrapped within discrete vesicles—macropinosomes [111]. This mechanism is instrumental for the internalization of large aggregates and cationic peptide vectors that exceed the size thresholds of other endocytic pathways (Figure 11).

#### 3.1.2. Impact of Peptide Properties on Uptake Efficiency

The precise topology and amino acid composition of peptides dictate their secondary structure, charge distribution, and amphiphilicity, thereby governing membrane interaction modes and uptake kinetics.

Arginine exhibits superior membrane translocation capabilities compared to Lysine. Although both carry a net positive charge at physiological pH, the guanidinium group on the Arg side chain confers a unique chemical advantage. Unlike the monodentate hydrogen bonding of the Lys amino group, the guanidinium moiety forms high-energy, stable bidentate hydrogen bonds with phosphate, sulfate, and carboxylate groups on the cell membrane [109,112]. This synergistic combination of electrostatic attraction and hydrogen bonding renders Arg-rich peptides significantly more affinable to membranes than other cationic variants, with uptake efficiency correlating directly with Arg density [1,42,108,112].

**Figure 12:**
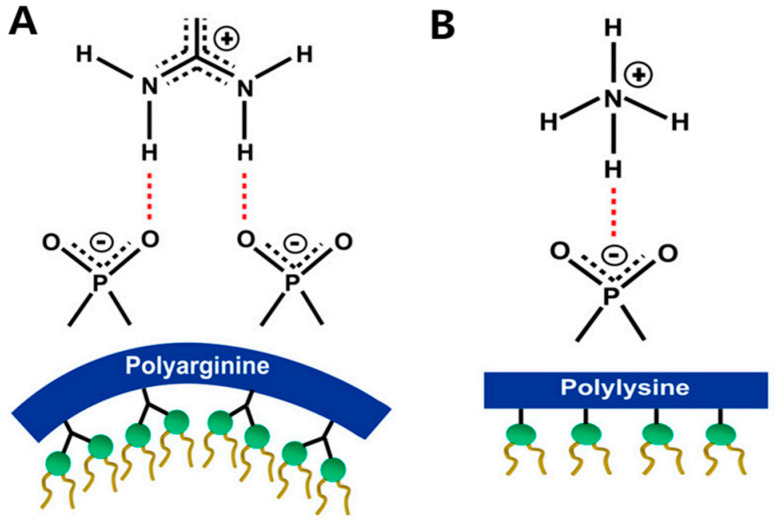
Bidentate hydrogen bonding of the arginine guanidinium group (**A**) and monodentate hydrogen bonding of the lysine amino group (**B**). Adapted from Ref. [116].

While single peptide-receptor interactions are often weak, displaying multiple peptides on a nanocarrier surface engenders a “multivalent effect.” Multivalent display facilitates simultaneous binding to multiple surface receptors, exponentially increasing avidity. Investigations demonstrate that branched TAT peptides, relative to their monomeric counterparts, achieve orders-of-magnitude enhancements in both endocytic uptake and transfection efficiency [107,113,114,115] (Figure 12).

### 3.2. Endosomal Escape Mechanisms

The proton sponge effect represents a pivotal paradigm in mediating endosomal escape mechanics. In the engineering of short peptide vectors, the strategic integration of histidine-rich motifs is employed to maximize this physicochemical phenomenon. The imidazole side chain of histidine exhibits a dissociation constant pKa situated precisely between the physiological extracellular environment and the acidic endosomal lumen. Consequently, histidine residues function as intrinsic pH-responsive toggles: retaining electrical neutrality within the extracellular matrix while undergoing rapid protonation upon internalization into the acidic endosome, a process that sequesters a substantial magnitude of protons [117,118,119]. To counteract this buffering capacity and sustain acidification, vacuolar proton pumps drive a continuous influx of protons; concurrently, to preserve electroneutrality, Cl^−^ passively diffuses into the lumen. This ionic accumulation precipitates a surge in intra-endosomal solute concentration, establishing significant osmotic pressure. The resulting osmotic gradient drives a massive influx of water molecules, inducing osmotic swelling that ultimately compromises membrane integrity, leading to endosomal lysis and the cytosolic liberation of the genetic cargo [117].

Membrane disruption constitutes a direct physical escape trajectory adopted by the majority of amphipathic peptides, a strategy predicated on the structural compromise of the endosomal lipid bilayer [120,121]. Functioning analogously to “pH-responsive molecular needles,” the lytic efficacy of these peptides is strictly governed by an acidity-induced conformational transition from a random coil to an α-helical architecture, a thermodynamic shift that drives peptide folding and subsequent membrane insertion [122]. A critical reorientation event typically follows, wherein the peptide axis shifts from a transmembrane alignment to an in-plane configuration parallel to the membrane surface. And this interfacial rearrangement fundamentally destabilizes membrane integrity [118,123]. Mechanistically, the hydrophobic face of the helix intercalates into the acyl chain core of the phospholipid bilayer, while hydrophilic residues project toward the aqueous pore lumen. This self-assembly generates transmembrane channels in which the lipid monolayers curve continuously to line the pore wall [117,124]—a topology that dissipates transmembrane ionic gradients while simultaneously establishing a physical conduit for the cytosolic translocation of genetic cargo [125].

**Figure 13:**
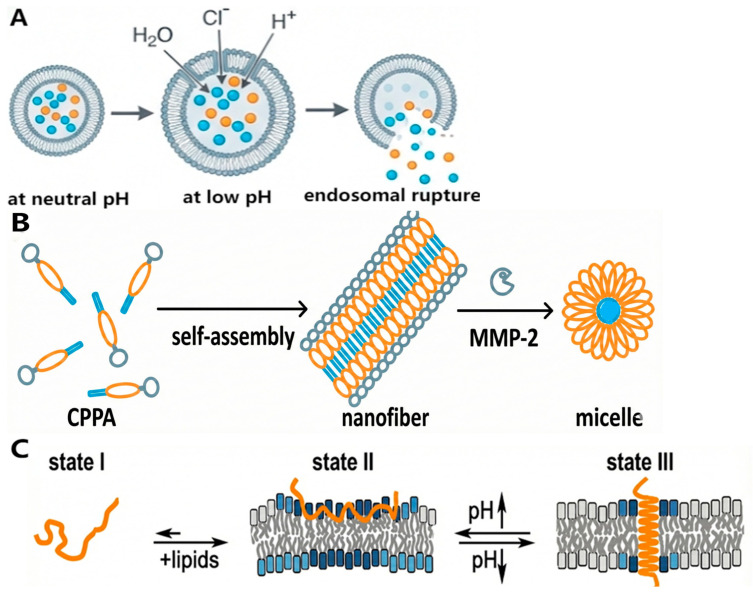
Schematic representation of major endosomal escape mechanisms for short peptide carriers: (**A**) Osmotic swelling and rupture induced by the proton sponge effect. (**B**) Enzyme-triggered morphological transition of assemblies. (**C**) Membrane insertion and disruption mediated by a pH-induced conformational transition. Adapted from Refs. [116,122,124].

While the high concentration of diverse hydrolases within endolysosomal compartments traditionally presents a degradative barrier to gene therapeutics, rational vector engineering exploits this enzymatic environment as a potent stimulus for escape. Leveraging superior biocompatibility and substrate specificity, enzyme-responsive peptide assemblies capitalize on the dysregulated expression profiles and differential enzymatic concentrations characteristic of pathological tissues or specific organelles. Consequently, these systems exhibit a selectivity profile that markedly surpasses that of vectors relying solely on general physical stimuli such as pH or thermal gradients [126,127]. The underlying mechanism entails the catalytic cleavage or formation of specific covalent bonds, which precipitates a programmed structural reconfiguration of the supramolecular assembly [126]. Within enzyme-rich microenvironments such as the lysosome, the vector transitions from a kinetically stable carrier state into a membrane-lytic active species, thereby facilitating efficient endosomal escape [127,128,129] (Figure 13).

### 3.3. Intracellular Release and Expression of Genetic Material

#### 3.3.1. Vector Disassembly Mechanisms Within the Cytoplasm

Successful translocation into the cytoplasm constitutes merely the initial phase of the intracellular journey. To realize therapeutic functionality, the genetic payload must be effectively liberated from the compact confinement of the short peptide vector. A central paradox in gene vector engineering involves resolving the stability-release dilemma, and vectors must exhibit inertness during systemic circulation yet undergo rapid activation upon cellular entry [130,131,132]. The incorporation of cysteine residues into peptide sequences transmutes the extracellular/intracellular chemical potential gradient into structural stability. Under oxidative extracellular conditions, thiol oxidation yields covalent disulfide bridges (-S-S-), functioning as molecular “locks” that immobilize the assembly [94,132]. This crosslinked disulfide network endows the complex with superior colloidal stability and resistance to serum interference, effectively mitigating premature leakage of the genetic cargo prior to target localization [131,132].

Upon cytosolic entry, elevated glutathione concentrations trigger the reductive cleavage of disulfide bonds via thiol-disulfide exchange reactions, precipitating vector disassembly—a mechanism dictated by the specific architectural archetype. For coacervates, the disintegration of the crosslinked network reduces local peptide concentrations below the phase separation threshold, inducing spontaneous dissolution and cargo release [131,132]. Conversely, in certain co-assembled nanoparticles, disulfide cleavage transforms hydrophobic tert-butyl disulfide moieties into hydrophilic cysteine residues; this disruption of the hydrophilic-hydrophobic equilibrium instigates instantaneous polymer disassembly, a process highly synergistic with the competitive displacement of the cargo by intracellular polyanions [133]. Since the reduction process weakens the binding affinity between the vector and DNA, negatively charged cytosolic macromolecules can readily displace the DNA from the loosened peptide structure. Indeed, research elucidates that efficient gene decompression is achievable only through the concerted action of reducing agents and polyanions [134].

**Figure 14:**
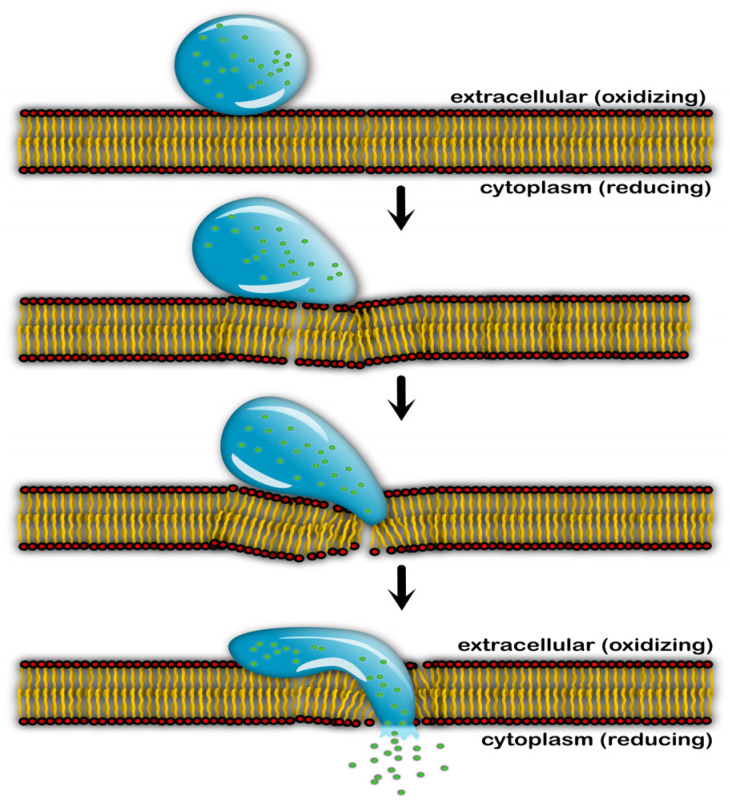
Redox-triggered cytosolic disassembly and cargo release. Adapted from Ref. [132].

For payloads requiring transcriptional processing, the nuclear envelope represents the final physical barrier. Lacking intrinsic active transport mechanisms, the majority of synthetic vectors rely on passive entrapment within daughter nuclei following mitotic envelope breakdown. This reliance results in a stark disparity in transfection efficiency between rapidly dividing and non-dividing cell populations [36]. In quiescent cells with intact nuclear envelopes, the nuclear pore complex serves as the sole conduit for macromolecular entry, yet it imposes a size exclusion limit of approximately 40 nm [131,135] (Figure 14).

To circumvent this, nuclear localization signals are widely integrated [136]. Addressing the NPC size bottleneck, recent dynamic modulation strategies have proven particularly elegant. For instance, photo-stimulation can induce a morphological transition from nanofibers to nucleotropic nanoparticles [135]. Conversely, for therapeutics targeting cytoplasmic sites [137], successful endosomal escape marks the culmination of the delivery process.

#### 3.3.2. Arrival of Released DNA/RNA at the Site of Action and Functional Expression

The primary phase of gene expression analysis focuses on transcriptional quantification and localization. While Illumina-based RNA-seq remains the gold standard for differential expression profiling due to its high throughput [138], long-read sequencing technologies such as PacBio and Nanopore have emerged as critical supplements for resolving full-length transcript isoforms [134]. Addressing the loss of spatial context inherent in bulk sequencing, RNAscope facilitates the leap from “quantification” to “localization”, coupled with digital image analysis, enabling precise detection of low-abundance genes. For validation, RT-ddPCR provides absolute quantification, though its sensitivity may be less robust than in situ hybridization when analyzing clinically degraded nucleic acids. Furthermore, the investigative paradigm is shifting from steady-state mRNA quantification to dynamic process analysis. Utilizing nascent RNA sequencing and non-invasive sensors like INTACT, researchers can now track transcriptional activity in real-time via blood assays without perturbing physiological states or compromising tissue integrity [139].

In contrast to transcriptional profiling, protein translation assessment directly reflects the functional realization of gene delivery [140]. During vector development, fluorescent proteins such as GFP are routinely paired with flow cytometry to calculate transfection percentages, while firefly luciferase utilizes wide-dynamic-range bioluminescence to precisely benchmark the efficacy of disparate delivery systems [133]. For endogenous verification, Western Blotting and ELISA remain the standard protocols for intracellular signaling and extracellular secretion analysis, respectively. However, translational omics is evolving toward higher resolution. Ribosome profiling (Ribo-seq) provides single-nucleotide resolution snapshots, while mass spectrometry combined with pulse labeling and the TRICK live-cell imaging system allow for the real-time visualization and quantification of nascent protein synthesis rates and single-molecule pioneer rounds of translation [140].

In research involving gene editing tools like CRISPR-Cas9, accurate assessment of DNA-level mutation efficiency is a prerequisite for interpreting expression variances. While the traditional T7E1 endonuclease assay is cost-effective, it frequently underestimates efficiency in samples with high mutation rates [141]. Sanger sequencing-based algorithms like TIDE or ICE improve quantification but retain bias in extreme editing ranges. In contrast, droplet digital PCR (ddPCR) offers superior resolution, making it particularly suitable for distinguishing between NHEJ and HDR repair products [142]. For the ultimate standard in accuracy and sequence detail, targeted NGS stands as the current premier tool. It not only corrects the quantitative biases of enzymatic methods but also reveals specific insertion/deletion patterns with high sensitivity [143], providing conclusive evidence for the safety and efficacy of gene editing.

## 4. Frontier Applications and Representative Studies of Short Peptide Gene Delivery Systems

This section systematically explores the latest advances in the application of short peptide vectors for delivering various mainstream gene therapy molecules. It focuses primarily on the physicochemical mechanisms and biological efficacies involved in RNA and CRISPR-Cas9 ribonucleoprotein (RNP) delivery. Finally, it extends this discussion to targeted delivery and combination therapy strategies. To provide a more intuitive overview of the current research landscape, the following table first summarizes representative applications, outlining the correspondence among specific target diseases, their corresponding gene therapy modalities, and the core short peptide delivery mechanisms (Table 6).

### 4.1. RNA Delivery

mRNA therapeutics have demonstrated transformative potential across infectious disease intervention, protein replacement therapies, and gene editing applications [137,144,149,150]. However, the physicochemical profile of mRNA—characterized by high molecular weight, significant hydrophilicity, and a dense negative charge density—renders transmembrane permeation inherently difficult [129,137]. To circumvent these barriers, the hPep3 system exemplifies superior physicochemical competence. The rational design strategy of this system relies on the precise modulation of peptide secondary structure. Researchers engineered a specific α-helical architecture that exploits spatial anisotropy to segregate functionality. One face of the helix is enriched with positively charged lysine residues to facilitate efficient electrostatic compaction of the anionic mRNA, while the opposing face displays hydrophobic leucine residues, establishing a hydrophobic core that stabilizes the nanoparticle assembly.

Subsequent incorporation of non-canonical amino acid modifications not only markedly enhanced the amphiphilicity of the peptide but also potentiated hydrophobic interactions, thereby substantially increasing the resistance of the nanoparticles to competitive displacement by polyanions. In non-human primate models, hPep3 assemblies successfully induced the expression of therapeutic-grade human erythropoietin [151]. Parallel developments in short peptide vectors have yielded similarly robust results. For instance, nanoparticles constructed from the WRAP5 peptide exhibit a favorable balance of physiological tolerance and controllable activation [152]. Furthermore, designs such as RALA have been instrumental in augmenting vaccine immunogenicity and potentiating specific T-cell responses [91,153].

Despite sharing a fundamental chemical architecture, small interfering RNA (siRNA) and miRNA execute distinct functional mechanisms: the former mediates the specific degradation of target mRNA, whereas the latter regulates gene networks via incomplete complementary binding. Both hold immense promise for gene therapy [147,154,155]. However, their clinical translation is impeded by severe physicochemical barriers, as hydrophilic polyanions, they are inherently refractory to biological membrane traversal [154,156]. A more intractable challenge lies in their topology. Unlike flexible plasmid DNA, short-chain RNAs are significantly shorter than their persistence length, causing them to behave as rigid rods in solution. This rigidity hinders entropic collapse, making them exceptionally difficult to condense using conventional polymers [157]. Compounded by a short in vivo half-life, the development of vectors capable of efficient condensation and cargo protection is critical [156].

Short peptide vectors targeting siRNA have evolved from simple cationic peptides into sophisticated, multifunctional assemblies [154]. To mitigate early-stage instability and cytotoxicity, cell-penetrating peptides have been integrated, leveraging hydrophobic domains to facilitate membrane fusion and endosomal escape [119,155].

Oleyl-conjugated WRH peptides improve hydrophobicity by adding fatty acyl chains and use the proton sponge effect of histidine to get out of endosomes quickly [147]. The canonical “WRAP” peptide series, optimized through specific tryptophan and arginine spatial arrangements, can rapidly “wrap” siRNA into homogeneous nanoparticles, significantly enhancing cellular uptake [119]. Additionally, the construction of poly-siRNA conjugates represents a novel strategy to augment stability; this polymerization not only reduces renal clearance but also markedly enhances liver targeting and gene silencing activity [158].

While sharing similar physicochemical properties, miRNA delivery necessitates heightened responsiveness to the tumor microenvironment. Addressing lung cancer, researchers designed the RPRIN nanoparticle system based on the mirror-image RGD-modified cationic peptide, RD24 [145]. This system exploits RGD for integrin receptor targeting and incorporates a matrix metalloproteinase-2 sensitive motif. The vector remains stable in circulation, but upon reaching the MMP2-rich TME or lysosome, proteolytic cleavage triggers peptide fragmentation and the rapid liberation of miRNA.

Similarly, hyaluronic acid-protamine complexes have been validated for effective targeting of CD44-overexpressing breast cancer cells, further broadening the application scope of multi-component self-assembling systems [148].

Transfer RNA (tRNA) therapy holds substantial promise, particularly for Anticodon Engineered tRNAs (ACE-tRNA) capable of suppressing premature termination codons (PTCs). In Xenopus oocyte models, ACE-tRNA delivery successfully rescued the G542X nonsense mutation in the CFTR gene. In murine skeletal muscle experiments, it repaired the NLuc-UGA reporter gene, inducing bioluminescence intensities that, at specific time points, exceeded those of the wild-type NLuc control without eliciting significant immune responses [159].

However, effective delivery remains impeded by unique structural constraints. Despite its small size, the highly rigid L-shaped tertiary structure of tRNA differs fundamentally from linear oligonucleotides. This rigidity renders it refractory to dense condensation by short peptides, often resulting in loose, unstable particles with poor cellular entry efficiency. Currently, LNPs and AAVs remain the dominant delivery modalities [160,161], making the development of peptide vectors for complex-structure short RNAs a key frontier for future breakthroughs.

Regarding ribosomal RNA (rRNA) delivery, this area represents a frontier characterized by both high difficulty and significant conceptual novelty. The delivery of rRNA offers potential cures for specific ribosomopathies and genetic disorders. Recent investigations have elucidated that certain positively charged short peptides, mimicking ribosomal protein tails, can undergo specific liquid–liquid phase separation with rRNA to form highly stable coacervates.

Although currently in the proof-of-concept stage, these findings implicate the feasibility of using short peptide assemblies for the delivery of macromolecular rRNA cargos [162].

### 4.2. Delivery of CRISPR-Cas9 Gene Editing Tools

The advent of CRISPR-Cas9 technology has fundamentally revolutionized the landscape of gene editing and therapeutics, distinguished by its precision, efficiency, and accessibility. This system is typically administered via three distinct modalities: plasmid DNA encoding Cas9 and sgRNA, a mixture of Cas9 mRNA and sgRNA, or pre-assembled ribonucleoprotein (RNP) complexes [163,164]. Among these, the RNP format offers the most significant clinical advantages. It bypasses the requisite intracellular transcription and translation processes, ensuring a rapid onset of action. Furthermore, the transient intracellular half-life of RNPs significantly mitigates off-target effects associated with prolonged Cas9 activity, while simultaneously abrogating the risk of insertional mutagenesis into the host genome [165,166,167]. Nevertheless, the safe and efficient translocation of this machinery into the nucleus of target cells remains a formidable barrier to clinical translation.

The delivery of RNP complexes is intrinsically challenged by their substantial molecular weight, net negative charge, and highly anisotropic charge distribution [165,166,167,168]. In response, peptide self-assemblies have emerged as premier vehicles, leveraging tunable charge densities and intrinsic membranolytic capabilities. To optimize encapsulation efficiency, researchers have delineated three primary strategies. First, co-assembly optimization focuses on the chemical refinement of modification motifs and peptide sequences. For instance, screening hydrophobic tails for hydrazide-bearing cationic peptides identified oleyl aldehyde as the optimal moiety, which forms stable ~270 nm nanoparticles via electrostatic co-assembly with Cas9 RNPs. Second, thermodynamic modulation involves thermal annealing of the sgRNA to potentiate its binding affinity for Cas9. Protocols involving the denaturation of crRNA and tracrRNA at 95 °C followed by renaturation foster stable RNA duplex formation, thereby optimizing SpCas9 RNP assembly and subsequent delivery efficiency. Third, the incorporation of anionic polymer stabilizers utilizes negatively charged polymers to interact with Cas9 RNPs; this effectively shields excessive positive charges, preventing aggregation and yielding compact, stable nanoparticles [169,170,171,172,173,174].

Following cellular internalization, failure to rapidly dissociate the RNP from the vector effectively sequesters its nuclease activity and precludes nuclear entry. Consequently, controlled cytosolic release constitutes a critical determinant of editing efficiency. Amphipathic systems such as PERC, RALA, and LAH5 primarily exploit pH sensitivity or conformational plasticity to compromise endosomal membrane integrity [164,175,176,177]. Conversely, vectors like (CR3)_3_C or HBpep-SP are engineered with disulfide linkages sensitive to the cytosolic reductive environment, utilizing high glutathione (GSH) concentrations to trigger vector disassembly [165,178]. Distinctly, the hPep3 system employs a silica core to adsorb proteins and peptides. It facilitates efficient gene editing by inducing rapid endosomal rupture, a process that releases the RNP into the perinuclear region and, critically, exposes the Cas9 nuclear localization signal (NLS) [167].

**Figure 15:**
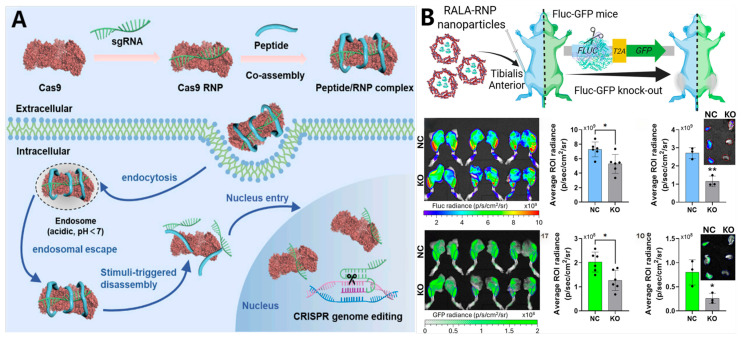
(**A**) Intracellular delivery mechanism. Peptide/RNP nanocomplexes form via electrostatic co-assembly. Following endocytosis, the acidic environment triggers endosomal escape, followed by cytosolic disassembly and nuclear entry for gene editing. (**B**) In vivo application in murine muscle. RALA-RNP nanoparticles injected into the tibialis anterior muscle successfully knocked out the Fluc-GFP reporter. Imaging analysis confirms significantly reduced bioluminescence in the knockout group compared to controls. * denotes significance *p* < 0.05, ** *p* < 0.01 as determined by an unpaired students *t*-test. Adapted from Refs. [165,175].

These peptide-mediated RNP delivery strategies have demonstrated exceptional efficacy. The optimized hPep3 system achieved adenine base editing efficiencies of up to 90% in HEK293T cells while maintaining robust editing levels in iPSCs and primary muscle stem cells [167]. In clinically critical primary T cells, the PERC system attained knockout efficiencies of 50–80% for Cas9 and >90% for Cas12a, exhibiting significantly reduced cytotoxicity and transcriptomic perturbation compared to electroporation [176]. The (CR3)_3_C vector achieved a gene editing efficiency of 33.8%, effectively inducing DNA double-strand breaks, verified by significant fluorescence signal induction. Furthermore, the RALA peptide demonstrated gene knockout yields in primary mesenchymal stem cells superior to commercial liposomes and was successfully applied to in vivo muscle editing [175]. The LAH5 system exhibited broad adaptability across various human cell lines, inducing targeted mutations in HeLa, HEPG2, and primary fibroblasts [164]. Notably, HBpep-SP-mediated delivery of Cas9 RNP achieved an indel frequency of 25.6%, markedly outperforming the 15.1% efficiency of the commercial reagent Lipofectamine CRISPRMAX [178] (Figure 15).

### 4.3. Targeted Delivery and Combination Therapy

#### 4.3.1. Achieving Organ or Cell-Specific Targeting via Ligand Integration

Monotherapeutic gene delivery often proves insufficient for addressing complex pathological landscapes. By endowing short peptide self-assemblies with active targeting capabilities and engineering them as versatile platforms for the co-delivery of genetic and chemotherapeutic agents, an integrated “recognition-attack-synergy” therapeutic paradigm can be realized.

In systemic administration, achieving site-specific nanoparticle accumulation remains a formidable challenge. While passive targeting relies on the Enhanced Permeability and Retention effect for tumor accumulation, its therapeutic efficacy is frequently compromised by significant intratumoral heterogeneity and dense stromal barriers [179,180]. Active targeting strategies, achieved via the surface decoration of nanocarriers with specific ligands, confer precise lesion recognition capabilities [181]. In the context of short peptide self-assembly, solid-phase peptide synthesis enables the efficient covalent integration of targeting motifs [182]. This technological advance shifts the delivery mechanism from inefficient passive diffusion to specific ligand-receptor interactions and subsequent receptor-mediated endocytosis [183]. Compared to non-targeted counterparts, this mechanism significantly amplifies cellular uptake efficiency and the therapeutic index.

The RGD sequence serves as the paradigmatic example, exhibiting specific recognition for integrin receptors [180]. These receptors are highly overexpressed on tumor neovasculature endothelia and various solid tumor cell surfaces, yet remain scarce in healthy tissue [180,181]. This differential expression endows RGD vectors with “dual-targeting” competence: the capacity to inhibit angiogenesis by targeting endothelial cells—thereby severing nutrient supply—while simultaneously mediating direct cytotoxicity against tumor cells [180,184].

To further potentiate efficacy, structural optimization strategies are widely employed. In contrast to linear sequences, which are conformationally flexible and susceptible to degradation, disulfide-cyclized RGD (cRGD) possesses superior structural rigidity [180]. This rigid conformation facilitates a more precise fit within the receptor binding pocket, significantly enhancing binding avidity and serum stability, thereby ensuring robust recognition within complex physiological environments.

#### 4.3.2. Co-Delivery of Chemotherapeutics and Gene Drugs for Synergistic Therapy

Carcinogenesis and tumor progression involve intricate signaling networks; consequently, monotherapies frequently precipitate the activation of compensatory pathways or the emergence of drug resistance. Combination therapy aims to achieve synergistic tumor eradication via distinct mechanisms [185]. The unique amphipathic architecture of short peptide self-assemblies enables the concurrent encapsulation of hydrophobic chemotherapeutics within a hydrophobic core and the adsorption of anionic genetic cargo onto hydrophilic surfaces or interlayers, thereby establishing an integrated co-delivery system.

In aqueous environments, hydrophobic amino acids or lipid tails function as hydrophobic domains, aggregating via hydrophobic interactions and π-π stacking to form a dense core [182]. This hydrophobic microdomain acts as a “reservoir,” facilitating the efficient physical entrapment of insoluble small molecules such as paclitaxel (PTX) or doxorubicin (DOX). For instance, the P17 lipopeptide can successfully load DOX via this mechanism [180].

Conversely, the hydrophilic shell is composed of cationic residues that utilize electrostatic interactions to densely condense and adsorb highly anionic genetic payloads (siRNA, pDNA, or ASO) [181,182,184]. Song et al. developed an RGD-functionalized methionine dialkylated peptide, which employs arginine sequences to co-assemble with Bcl-2 ASO, yielding monodisperse spherical complexes with uniform particle size [182].

This dual-loading architecture not only provides a physical barrier protecting genetic cargo from enzymatic degradation but also enhances cellular uptake via ligand integration [181]. Crucially, this system ensures the synchronous delivery of both therapeutic agents to the same cell at a precise stoichiometric ratio. This fundamentally overcomes the “spatiotemporal mismatch” caused by the divergent pharmacokinetics (PK) of free drugs, thereby realizing synergistic antitumor effects. For example, multidrug resistance (MDR), a primary bottleneck in oncology, often stems from reduced drug uptake and the upregulation of anti-apoptotic proteins [183]. To surmount this dual barrier, peptide-functionalized nanomedicine strategies have emerged, aiming to bridge the gap from simple “chemosensitization” to highly efficient “synergistic therapy” via co-delivery [183,186].

## 5. Challenges and Future Perspectives

### 5.1. Major Challenges

Despite the transformative potential of short peptide assemblies in drug delivery and disease therapeutics, their clinical translation is currently circumscribed by four formidable barriers: in vivo stability, targeting specificity, large-scale manufacturing and quality control, and immunogenicity coupled with long-term toxicity.

In vivo, short peptide vectors confront an environment replete with proteolytic enzymes; the native amide bond between amino acids serves as a primary substrate for serum proteases. Although supramolecular self-assembly confers a degree of steric shielding, surface-exposed residues remain vulnerable to enzymatic degradation. Consequently, unmodified peptides are rapidly identified and cleaved within the systemic circulation, a liability that severely restricts clinical utility [187,188,189]. To augment systemic circulation half-lives, molecular engineering strategies have been employed, including the incorporation of non-canonical amino acids, peptoid backbones, or terminal modifications to mitigate enzymatic recognition [190]. Topologically, cyclization strategies significantly reduce the exposure of proteolytic cleavage sites by eliminating free termini and enhancing conformational rigidity [116,191]. Furthermore, supramolecular “locks” mediated by metal ion coordination can mechanically reinforce the assembly structure [187]. Concurrently, the construction of a surface hydration layer via PEGylation exploits steric hindrance to block protease access and minimize immune clearance, while lipidization of short peptides further prolongs systemic retention [188,192,193].

Beyond stability, achieving precise lesional accumulation while minimizing off-target toxicity constitutes a critical bottleneck. Although peptide vectors are frequently touted for superior specificity compared to small molecules, the “off-target” phenomenon remains a primary toxicological driver of clinical attrition within complex physiological environments. Many peptide receptors, while overexpressed in pathological tissues, maintain basal expression levels in healthy organs [183,194,195]. In response, multi-dimensional precision targeting strategies have been established. The conjugation of high-affinity ligands combined with conformational constraints via cyclization can markedly potentiate receptor-mediated endocytosis and binding avidity [43,191]. Moreover, constructing intelligent responsive systems that leverage pathological microenvironmental cues to trigger site-specific phase transitions or cargo release represents a key pathway to reducing systemic toxicity [187,191]. When integrated with “targeting-plus-penetration” chimeric peptides, biomimetic cell membrane camouflaging, and peptide-drug conjugate designs, these approaches are facilitating a paradigm shift from inefficient “passive diffusion” to high-efficiency “precision strikes” [43,187].

The transition from milligram-scale laboratory synthesis to kilogram-scale industrial manufacturing unearths a sharp conflict between chemical synthesis efficiency and environmental sustainability. While solid-phase peptide synthesis remains the dominant production modality, it generates substantial chemical waste [196].

Economic viability acts as a further constraint, with purification representing the most cost-intensive sector of the production line. Even impurity peptides differing by a single amino acid from the target sequence often exhibit nearly identical chromatographic behaviors. Separating such species via reversed-phase high-performance liquid chromatography necessitates prolonged run times and massive consumption of acetonitrile mobile phases, resulting in excessive energy expenditure. Particular vigilance is required regarding “aggregates” and “conformational isomers”. These high-order structural impurities are not only arduous to detect but are also intimately linked to immunogenic responses [169,197,198]. Consequently, the development of green synthetic methodologies is imperative.

Although short peptide assemblies generally exhibit superior biocompatibility relative to proteins, their potential immunogenicity and long-term toxicity remain significant translational hurdles. Exogenous sequences, chemical modifications, or specific nanostructures may be flagged as “non-self” antigens, precipitating therapeutic neutralization, altered pharmacokinetics, or hypersensitivity reactions [43,199]. Traditional PEGylation strategies face severe headwinds, and the induction of anti-PEG antibodies can lead to the ABC phenomenon, diminishing the efficacy of repeat dosing [190,192,200].

**Figure 16:**
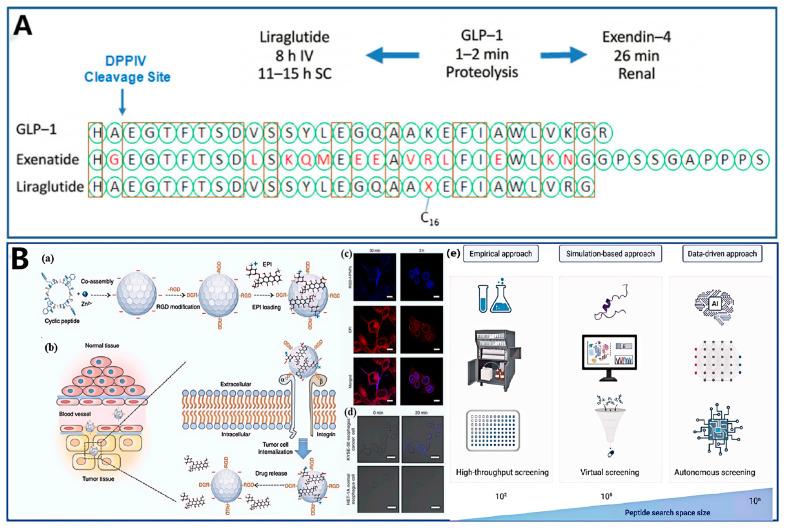
(**A**) Chemical modifications for stability. Strategies such as lipidation and amino acid substitution significantly extend the in vivo half-life of peptide drugs. (**B**) (**a**)Supramolecular assembly and active targeting. Metal coordination and cyclic topology stabilize the nanostructure, (**b**)while RGD ligands enable precise tumor delivery. (**c**) Cellular Uptake: Fluorescence intensity increased over time, confirming effective drug internalization. (**d**) Targeting Specificity: The drug selectively accumulated in cancer cells but not in normal cells, validating precise targeting. (**e**) Evolution of design paradigms. The discovery methodology is shifting from empirical high-throughput screening to data-driven, AI-assisted rational design. Adapted from Refs. [187,188,202].

Furthermore, the long-term metabolic fate and cumulative toxicity of synthetic impurities, stabilizing non-canonical amino acids, crosslinkers, or metal ions remain under-characterized [190,191]. Addressing these challenges necessitates a dual approach comprising low-immunogenicity design and a multi-tiered evaluation hierarchy. On the design level, strategies include the bioinformatic excision of T-cell epitopes, the adoption of biomimetic sequences for “de-immunization” [190,199], and the exploration of zwitterionic polymers, glycosylation, or fatty acid conjugation as PEG alternatives [191], alongside physical shielding of recognition sites via cyclization or nano-encapsulation. Regarding evaluation, a comprehensive “In Silico-In Vitro-In Vivo” screening platform must be established. This closed-loop “design-assess” strategy is essential for guiding the development of high-purity, low-toxicity therapeutics and ensuring long-term clinical safety [192,199,201] (Figure 16).

More fundamentally, however, the development of short-peptide nanodrugs is hindered by systemic flaws and mismatches that extend beyond these technical limitations. These issues severely bottleneck their real-world medical application. Critically speaking, the current R&D paradigm is trapped in a dilemma between idealized designs and harsh clinical realities [43].

Existing animal models simply cannot replicate the extreme complexity of human physiology [43,199]. Consequently, assemblies that perform flawlessly in vitro often face a rude awakening once introduced into the human body [43,191]. Confronted with the dual threats of high tumor heterogeneity and receptor non-exclusivity, they are highly prone to off-target toxicity and plummeting efficacy [187]. To bypass absorption hurdles, developers often resort to strategies such as adding enzyme inhibitors, utilizing highly lipophilic prodrugs, or relying on notoriously sluggish lymphatic transport [189]. These workarounds act as a double-edged sword; rather than tackling physiological barriers at their root, they introduce new, uncontrollable risks like abnormal protein metabolism and non-specific binding. Furthermore, the reliance on complex administration routes, such as long-acting injections or inhalation, only exacerbates the ongoing struggle with poor patient compliance and unpredictable absorption [189,190].

On the manufacturing front, the shift toward “green chemistry” risks introducing entirely novel, uncharacterized impurities. Yet, regulatory bodies currently lack unified threshold guidelines for peptide impurity profiling, leaving a massive blind spot in safety evaluations [197]. Couple this with synthesis costs that dwarf those of small-molecule drugs by orders of magnitude, extreme inter-individual variability in pharmacokinetics, and the sobering precedent of existing products that are clinically effective but commercially unviable [188]. As a result, short-peptide nanodrugs carry a dauntingly high commercial risk for trial and error.

Ultimately, unless the industry can fundamentally break its reliance on animal models, establish clear impurity regulations, and completely overhaul the cost–benefit paradigm, short-peptide assemblies will struggle to bridge the chasm separating laboratory novelties from viable clinical therapeutics.

### 5.2. Future Directions

In light of the significant hurdles mentioned earlier, short peptide vector research is transitioning from “passive adaptation” to “active intelligence.” Future advancements transcend simple chemical derivatization, and they conceptualize peptides as programmable bio-informational entities, reconfiguring the essential principles of drug delivery via intelligent, biomimetic, and cohesive architectural frameworks.

Vector engineering is transcending single-stimulus responsiveness to establish intelligent dynamic systems capable of integrating multi-dimensional signals. By orchestrating synergistic mechanisms responsive to pH, enzymatic activity, redox potentials, and photothermal cues, the specificity and spatiotemporal controllability of delivery are significantly potentiated [97]. Such vectors surpass the rudimentary function of cargo release to realize in situ structural evolution and self-sorting [203]. For instance, glutathione (GSH)- induced assemblies can morph from nanorods to nanoribbons to synergistically induce apoptosis, while methionine-containing assemblies undergo reactive oxygen species (ROS)-triggered gel-to-sol transitions to scavenge excess H_2_O_2_, thereby conferring cytoprotection [204]. At the frontier, enzyme-specific induction of liquid–liquid phase separation is being exploited to generate biomimetic coacervates [51]. These structures not only efficiently encapsulate cargo but also sequester critical enzymes to modulate metabolic flux, demonstrating significant potential for active intracellular functionalization [205].

Addressing the intricate structure-function relationships between peptide sequences and microenvironmental responsiveness, Artificial Intelligence (AI)-assisted rational design has emerged as a core engine accelerating vector development. Leveraging Machine Learning and Large Language Models, researchers can precisely predict self-assembly phase behaviors under varying conditions, optimize linker stability in peptide-drug conjugates, and refine payload screening, thereby circumventing the inefficiencies of traditional trial-and-error methodologies. This data-driven paradigm propels peptide vectors from passive transport vehicles to “intelligent nanomachines” integrating environmental sensing, logical computation, and structural deformability—roles pivotal to the future of precision medicine [206,207,208].

In the realm of functional biomimicry, design efforts focus on precisely recapitulating the sequential breaching of physiological barriers—spanning membrane penetration, endosomal escape, and nuclear trafficking—characteristic of viral infection. By incorporating perfluoroalkyl chain modifications or constructing “enveloped virus replicas,” researchers exploit the unique hydrophobicity and membrane-fusing capabilities of PFA to mimic mechanisms such as caveolae-mediated endocytosis for efficient cellular entry [209].

This holistic biomimetic strategy offers a novel trajectory for developing high-efficiency, low-toxicity, and functionally customizable gene and drug delivery systems. Concomitant with the evolution of precision medicine, peptide-based nanomaterials are advancing toward “Theranostic” platforms integrating diagnostic and therapeutic functionalities. Through chemical conjugation or physical encapsulation, targeting peptides are combined with photothermal agents such as copper sulfide, melanin, or gold nanostars. These systems not only execute photothermal therapy [210] but also enable precise localization guided by photoacoustic imaging, MRI, or near-infrared fluorescence imaging, truly realizing a “see-and-treat” paradigm. To transcend single-mode limitations, future platforms will deeply integrate multimodal imaging to endow vectors with deep tissue penetration and high-resolution boundary delineation capabilities, enhancing diagnostic and navigational efficiency for complex lesions like brain tumors via real-time tracking of gene payload distribution [97].

At the same time, AI is changing the way research and development works. Most of the PDC projects that have recently entered clinical trials used AI-assisted design [207]. Algorithms like AlphaFold and RFdiffusion make it easier to build new peptides and optimize linkers. This gets rid of the need for trial and error at the source and cuts down on clinical failure rates by a huge amount.

Ultimately, bridging the gap between the laboratory bench and the clinical bedside requires addressing engineering scalability. Future collaborative endeavors must shift towards the creation of sustainable, manageable industrial platforms, exemplified by the use of microfluidics for the economical and reproducible production of nanovectors [97]. Additionally, the academic and industrial sectors must collaboratively develop animal models and evaluation standards that more accurately represent clinical realities, thereby facilitating a seamless transition from proof-of-concept to clinical application.

## Figures and Tables

**Table 1:** Comparison of the advantages and disadvantages of gene delivery carrier systems [29,38,39,40].

Carrier Type	Advantages	Disadvantages
Viral vectors	Powerful in vivo transfection efficiency. Long-term transgene expression.	Immune response. Inefficient transduction. Size limitations. Low gene loading capacity.
Lipid-based nanocarriers	High load capacity. Degradability. Easy to modify structure and charge.	Toxicity at high doses.
Natural polymer-based nanocarriers	Biocompatible and biodegradable. Minimal immunogenicity. Efficient condensation and protection of genetic material. Stimuli-responsive degradation.	Limited gene loading capacity. Lower transfection efficiency compared to viral vectors. Variable batch-to-batch consistency. Susceptibility to enzymatic degradation in vivo.
Synthetic polymer-based nanocarriers	Powerful gene chelation ability. Improved endosomal escape.	Cytotoxicity. Complex preparation.
Nanoclays	High loading capacity. Safety and cost-effectiveness. Payload protection.	Potential toxicity. Surface reactivity risks. Aggregation tendency.

**Table 2:** Clinical Application and Safety Assessment of Different Delivery Carriers.

Carrier Type	Toxicity and Safety	Target Diseases and Applications	Clinical Stage	Outcomes
Viral vectors	High immunogenicity [28]	Monogenic genetic diseases, cancer/solid tumors, hematologic malignancies [27]	Multiple products have been approved for marketing [27]	Highest in vivo transfection efficiency
Lipid-based nanocarriers	Toxic at high doses [29]	Infectious disease vaccines, metabolic diseases, solid tumors/cancer [27,35,41]	Approved for marketing and widely applied [27]	Successfully clinically translated non-viral vectors
Synthetic polymer-based nanocarriers	Relatively high cytotoxicity	Solid tumors/cancer, cystic fibrosis [29]	Some have entered Phase I/II clinical trials, and most are still in preclinical stages	Strong gene integration capability, high challenge for in vivo translation
Nanoclays	Extremely low toxicity for oral or local administration [39]	Bone and cartilage regenerative medicine/tissue engineering, neurodegenerative diseases, and local tumors [40]	Preclinical stage [40]	Extremely high encapsulation efficiency
Short peptide self-assemblies	Extremely low, good biocompatibility	Tumor/cancer therapy, tissue engineering and regenerative medicine, theranostics	Preclinical stage	Difficulties exist in clinical translation, but highly promising

**Table 3:** Characteristics Comparison of Different Delivery Carrier Types.

Carrier Type	Particle Size	Surface Charge	Transfection Efficiency
Viral vectors	Approx. 70–100 nm [27]	Depends on its specific viral capsid or envelope glycoproteins.	Extremely high. Most efficient in vivo vector; infects both dividing and non-dividing cells [27,28,29].
Lipid-based nanocarriers	Usually < 200 nm [21]	Traditional lipids carry a constant positive charge; ionizable lipids are neutral at physiological pH and positively charged in an acidic environment.	Relatively high. Has shown excellent efficiency in the clinical delivery of siRNA [35].
Synthetic polymer-based nanocarriers	Mostly between 50–300 nm [30,36]	High positive charge density. Rich in cationic groups.	Relatively high. Uses “proton sponge effect” to boost endosomal escape and transfection [29,36].
Nanoclays	Various sizes [38]	Varies depending on the type.	Good. Protects nucleic acids from degradation and uses electrostatic interactions to boost cellular uptake [38,40].
Short peptide self-assemblies	Various sizes, particle size is adjustable [43]	Depends on the amino acid composition of the peptide and the environmental pH value.	High.

**Table 4:** This table illustrates four types of molecular interactions—electrostatic interactions, hydrogen bonding, hydrophobic interactions, and π-π stacking—and lists the corresponding key amino acids, functional groups, and their specific functions.

Interaction Type	Key Amino Acids and Groups	Function in Gene Delivery
Electrostatic Interaction	Arg, Lys, Phosphate Backbone [54]	DNA Condensation; Charge neutralization; Facilitates cell membrane adsorption [55]
Hydrogen Bonding	Amide bonds, Water molecules	Forms secondary structures (β-sheets and α-helices); Stabilizes hydrogel networks [56]
Hydrophobic Interaction	Ala, Val, Leu, Ile, Phe	Drives the formation of micelle and vesicle cores; Determines CMC and overall stability [57,58]
π-π Stacking	Phe, Trp, Tyr	Provides structural rigidity; Enhances thermal stability of the carrier [54]

**Table 5:** Summary of loading mechanisms, advantages, and limitations of gene vectors.

Structure Type	Gene Loading Mechanisms	Advantages	Limitations
Nanotubes/Nanofibers	Surface wrapping/Electrostatic adsorption [82]	High specific surface areaMimics viral structure [77]	Difficult nuclear entry [81]
Nanovesicles	Encapsulation within the internal aqueous phase	Complete protection of cargo; capable of stimuli-responsive release	Structural stability is sensitive to the environment;complex preparation process [70]
Hydrogels	Network entrapment/Electrostatic adsorption	Localized delivery; long-term sustained release; excellent biocompatibility [92]	Restricted to local application; unable to target via systemic blood circulation [92]

**Table 6:** Examples of Gene Therapy Using Short Peptide Delivery Systems.

Target Disease	Cargo	Short Peptide Delivery System	Therapeutic Strategy
Lung Cancer	CRISPR-Cas9 System	ADGN Peptide	Knockout [144]
LUAD	miRNA	RD24 Peptide	Expression Regulation [145]
Fatal Viral Encephalitis	siRNA	RVG-9R Chimeric Peptide	Silencing [146]
Breast Cancer, Ovarian Cancer	siRNA	Oleyl-WRH Peptide	Silencing [147]
Prostate Cancer, Melanoma, Rabies.	mRNA	Protamine	Expression [137,148]

## Data Availability

No new data were created or analyzed in this study. Data sharing is not applicable to this article.
